# Designing Modular Cell-free Systems for Tunable Biotransformation of l-phenylalanine to Aromatic Compounds

**DOI:** 10.3389/fbioe.2021.730663

**Published:** 2021-07-28

**Authors:** Chen Yang, Yushi Liu, Wan-Qiu Liu, Changzhu Wu, Jian Li

**Affiliations:** ^1^ School of Physical Science and Technology, ShanghaiTech University, Shanghai, China; ^2^ Shanghai Advanced Research Institute, Chinese Academy of Sciences, Shanghai, China; ^3^ University of Chinese Academy of Sciences, Beijing, China; ^4^ Danish Institute for Advanced Study (DIAS) and Department of Physics, Chemistry and Pharmacy, University of Southern Denmark, Odense, Denmark

**Keywords:** cell-free systems, biocatalysis, biotransformation, value-added chemicals, synthetic biology

## Abstract

Cell-free systems have been used to synthesize chemicals by reconstitution of *in vitro* expressed enzymes. However, coexpression of multiple enzymes to reconstitute long enzymatic pathways is often problematic due to resource limitation/competition (e.g., energy) in the one-pot cell-free reactions. To address this limitation, here we aim to design a modular, cell-free platform to construct long biosynthetic pathways for tunable synthesis of value-added aromatic compounds, using (*S*)-1-phenyl-1,2-ethanediol ((*S*)-PED) and 2-phenylethanol (2-PE) as models. Initially, all enzymes involved in the biosynthetic pathways were individually expressed by an *E. coli*-based cell-free protein synthesis (CFPS) system and their catalytic activities were confirmed. Then, three sets of enzymes were coexpressed in three cell-free modules and each with the ability to complete a partial pathway. Finally, the full biosynthetic pathways were reconstituted by mixing two related modules to synthesize (*S*)-PED and 2-PE, respectively. After optimization, the final conversion rates for (*S*)-PED and 2-PE reached 100 and 82.5%, respectively, based on the starting substrate of l-phenylalanine. We anticipate that the modular cell-free approach will make a possible efficient and high-yielding biosynthesis of value-added chemicals.

## Introduction

Biotransformation is a green and sustainable approach for the production of valuable chemicals, pharmaceuticals, and materials, among others (Bornscheuer et al., 2012; Sheldon and Woodley, 2018; Wu and Li, 2018; Wang et al., 2021; Wu et al., 2021). For decades, microbial organisms have been predominantly engineered to synthesize those compounds of interest from cost-effective carbon sources like renewable biomass (e.g., cellulose and starch, etc.) and one-carbon feedstocks (e.g., formate and CO_2_, etc.) (Humphreys and Minton, 2018; Cotton et al., 2020; Ko et al., 2020). While cell-based biotransformation is a promising means for the production, efforts to design and engineer living organisms are constrained by slow design-build-test (DBT) cycles (Nielsen and Keasling, 2016). In addition, overexpression of a set of exogenous enzymes to perform *in vivo* chemical conversions imposes a strong metabolic burden on heterologous microbial hosts. Another key challenge in cellular production is the balance (conflict of resource allocation) between the cell’s survival objectives (i.e., native metabolism and growth) and the engineer’s production goals (i.e., synthesis of target products), making the final product yields in cellular systems often low and not satisfactory (Li and Neubauer, 2014; Wu et al., 2016). Therefore, designing and building efficient systems for biotransformation and maximization of product yields is highly desirable.

As a complement to cell-based approaches, cell-free systems have emerged as promising and powerful platforms for biomanufacturing (Bundy et al., 2018; Li et al., 2018; Swartz, 2018; Liu et al., 2019; Bowie et al., 2020; Silverman et al., 2020; Rasor et al., 2021). The unique feature of cell-free systems is that cell growth and product synthesis are separated, thus moving biosynthesis away from living cells and converting input resource/energy into desired products at high yields. Due to the open nature of cell-free systems, the reaction environment can be easily manipulated, directly accessed, and rapidly optimized. Furthermore, cell-free systems without cellular barriers can bypass transfer limitations and are more tolerant of toxic molecules (e.g., substrates, pathway intermediates, and final products) than living microbial cells. Recently, crude extract based cell-free protein synthesis (CFPS) systems were well developed and their applications have been expanded from single protein synthesis to multiple enzyme coexpression (Kwon et al., 2013; Li et al., 2016; Goering et al., 2017; Moore et al., 2021). The CFPS-expressed enzymes without purification can carry out *in situ* cascade biotransformation to synthesize a wide variety of molecules, such as *n*-butanol (Karim and Jewett, 2016), diketopiperazine (Goering et al., 2017), polyhydroxyalkanoate (Kelwick et al., 2018), styrene (Grubbe et al., 2020), 3-hydroxybutyrate (Karim et al., 2020), limonene (Dudley et al., 2020), l-theanine (Feng et al., 2021), and the complex natural product valinomycin (Zhuang et al., 2020). Notably, the yields of the CFPS-based production platform are often higher than *in vivo* production. For example, CFPS systems improved the production of styrene and valinomycin both by more than two times as compared to recombinant cellular systems (Grubbe et al., 2020; Zhuang et al., 2020).

Despite cell-free systems have many advantages and have been applied to reconstitute *in vitro* metabolic pathways for biotransformations, the reported complete pathways in CFPS systems were normally short with less than four enzymes. This is mainly because some full pathways are too long–with many enzymes–to express in a single-pot CFPS. To alleviate the tension of all enzyme coexpression in one pot, partial enzymes of the pathway can be individually expressed *in vivo* to prepare enzyme enriched cell extracts, which then can be used for CFPS to express the rest enzymes to assemble the full biosynthetic pathway (Dudley et al., 2020; Karim et al., 2020; Zhuang et al., 2020). However, this strategy is laborious and time-consuming that includes *in vivo* overexpression of each pathway enzyme, preparation of multiple cell lysates, and *in vitro* mix-and-match of lysate combination. As comparison to *in vivo* expression (days), CFPS systems can significantly reduce the expression time (hours) from DNA (plasmids) to functional enzymes. Therefore, with the rapid property of CFPS reactions, the overall time for enzymatic biotransformation will also be notably reduced if all enzymes of a long pathway can be coexpressed by CFPS.

Recently, we reported the use of CFPS to coexpress three enzymes for the bioconversion of styrene to (*S*)-1-phenyl-1,2-ethanediol with a high conversion rate (Liu et al., 2020). However, the substrate styrene is water-insoluble. Then, an aqueous-organic biphasic system was used to support the enzyme expression and the resulting cascade biotransformation. While this system worked well, an organic solvent like the toxic toluene was required to dissolve styrene, which is not a green or environmentally friendly approach. To avoid using organic solvents, a metabolic pathway is thus needed to synthesize styrene from possible water soluble substrates as an intermediate, which can be enzymatically converted to the downstream products. Such pathway is available by using three enzymes to convert l-phenylalanine (L-Phe) to styrene (Lukito et al., 2019). However, this solution will bring more enzymes to coexpress in one CFPS reaction and as a result coexpression of all enzymes might be problematic. Thus, a rational strategy is needed to solve the issue of multiple enzyme coexpression in a one-pot CFPS reaction.

**FIGURE 1:**
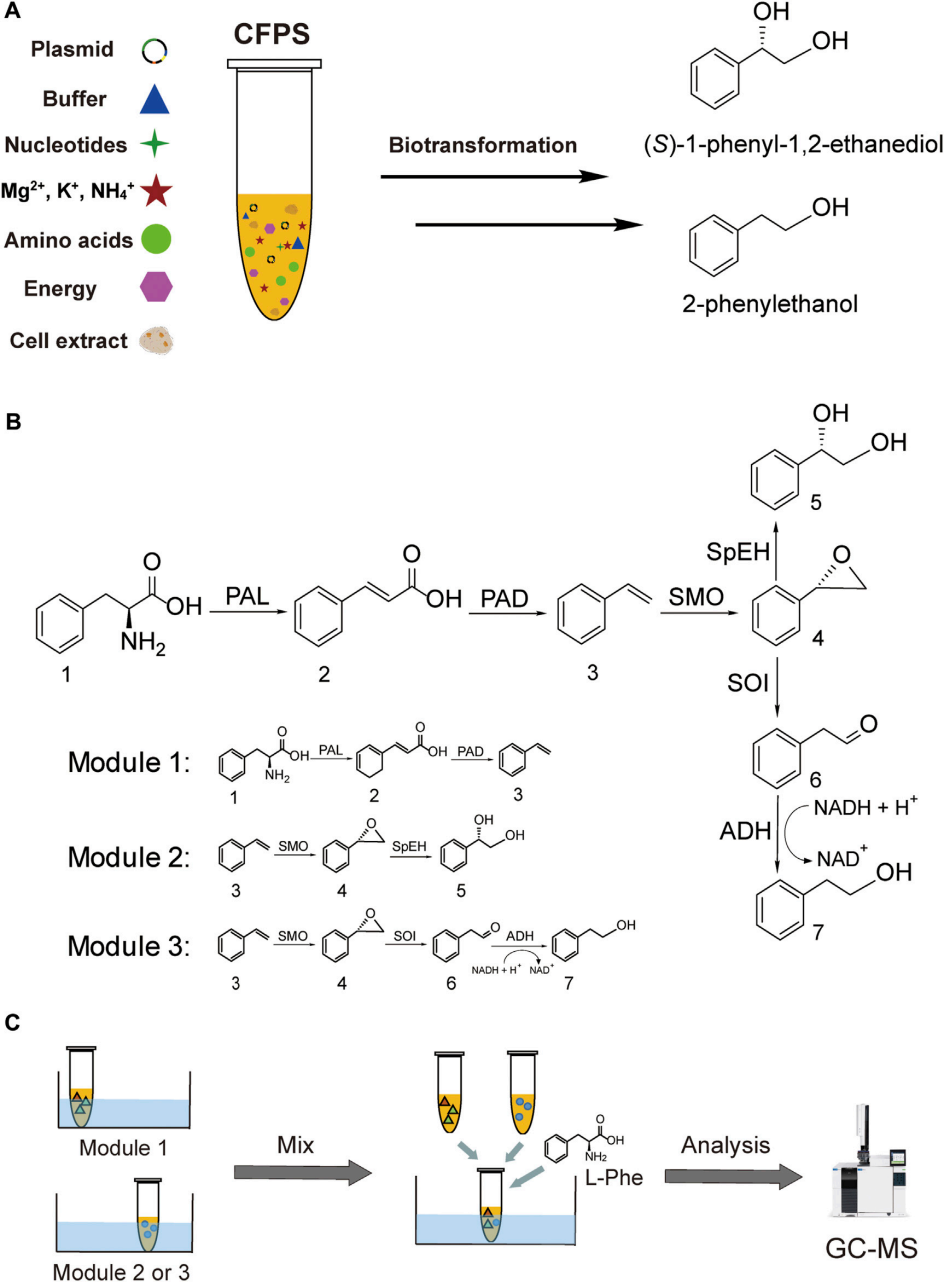
Cell-free synthesis of (*S*)-PED and 2-PE. **(A)** CFPS reaction and the synthesis of target chemicals. **(B)** Overall enzymatic pathways and designing of three modules. **(C)** Modular cell-free systems for the product formation. Abbreviations: CFPS, cell-free protein synthesis; PAL, phenylalanine ammonia lyase; PAD, phenylacrylic acid decarboxylase; SMO, styrene monooxygenase; SpEH, epoxide hydrolase; SOI, styrene oxide isomerase (also called StyC); ADH, alcohol dehydrogenase. Chemicals 1–7: 1, l-phenylalanine (L-Phe); 2, cinnamic acid; 3, styrene; 4, styrene oxide; 5 (*S*)-1-phenyl-1,2-ethanediol ((*S*)-PED); 6, phenylacetaldehyde; 7, 2-phenylethanol (2-PE).

In this work, we aim to address the challenge by designing modular cell-free systems for tunable biotransformation, which can be used to convert the same precursor L-Phe to two value-added aromatic compounds without using the water-insoluble substrate styrene (Figure 1). The first target compound is the chiral drug intermediate (*S*)-1-phenyl-1,2-ethanediol ((*S*)-PED) and the second one is the rose-like fragrance 2-phenylethanol (2-PE) (Peng et al., 2019; Wang et al., 2019). These two aromatic compounds share a common upstream intermediate styrene. To achieve our goal, here we design three CFPS modules. In the first module, an artificial pathway from L-Phe to styrene is constructed by coexpression of three enzymes, namely, phenylalanine ammonia lyase 2 (PAL2) from *Arabidopsis thaliana* (Cochrane et al., 2004) and phenylacrylic acid decarboxylase (PAD, containing two enzymes Fdc1 and Pad1) from *Aspergillus niger* (Payne et al., 2015). The second module uses styrene generated in the first module to synthesize (*S*)-PED, where another three enzymes are coexpressed to perform the cascade reaction. The related enzymes include styrene monooxygenase (SMO, consisting of two subunit enzymes StyA and StyB) from *Pseudomonas* sp. VLB120 (Panke et al., 1998) and epoxide hydrolase (SpEH) from *Sphingomonas* sp. HXN-200 (Wu et al., 2013). In the third module, three enzymes (StyA, StyB, and StyC) involved in the styrene degradation pathway (Panke et al., 1998) are employed to convert styrene to phenylacetaldehyde, which can be reduced to the final product 2-PE by endogenous alcohol dehydrogenases (ADHs). Our data suggest that the designer CFPS modules are efficient for long enzymatic cascade reactions, enabling high conversion rates of L-Phe to (*S*)-PED and 2-PE at 100 and 82.5%, respectively. Looking forward, we anticipate this work will expand the application space of cell-free systems by designing modular CFPS reactions to carry out long cascade biotransformations for efficiently synthesizing valuable chemicals when the whole-cell bioconversion remains difficult.

## Materials and Methods

### Chemicals

l-phenylalanine was purchased from Sigma-Aldrich (St. Louis, United States). Styrene, styrene oxide, (*S*)-1-phenyl-1,2-ethanediol, and 2-phenylethanol were purchased from Adamas (Shanghai, China). Cinnamic acid and phenylacetaldehyde were obtained from Aladdin (Shanghai, China). The plasmid miniprep kit was purchased from Sangon Biotech (Shanghai, China). All other chemical reagents were of the highest purity available.

### Strains and Cultivation Media

*Escherichia coli* DH5α was used for molecular cloning and plasmid propagation. *E. coli* BL21 Star (DE3) was grown for cell extract preparation to perform CFPS reactions. General cultivation of *E. coli* strains was carried out in the LB medium (10 g/L tryptone, 5 g/L yeast extract, and 10 g/L NaCl). The 2xYTPG medium (10 g/L yeast extract, 16 g/L tryptone, 5 g/L NaCl, 7 g/L K_2_HPO_4_, 3 g/L KH_2_PO_4_, and 18 g/L glucose, pH 7.2) was used to grow *E. coli* cells for cell extract preparation.

### Plasmid Construction

The plasmids pET28a-StyA, pET28a-StyB, and pET28a-SpEH used for the expression of StyA, StyB, and SpEH, respectively, were reported in our previous work (Liu et al., 2020). All other genes were codon optimized for *E. coli*, synthesized, and cloned into the plasmid pET28a by GENEWIZ (Suzhou, China), including *PAL2* (GenBank: AY303129), *Fdc1* (GenBank: XM_001390497), *Pad1* (GenBank: XM_001390495), and *StyC* (GenBank: AF031161). The gene *StyC* encodes a styrene oxide isomerase (StyC) in the styrene degradation pathway (Panke et al., 1998). To construct fused StyA and StyB, a flexible linker sequence [AS (GGGGS)_5_GAS] was inserted between the amino acid sequences of the two enzymes and cloned into pET28a, generating the plasmid pET28a-StyA-linker-StyB. All genes were inserted to the pET28a backbone between two restriction sites NdeI and SalI (see Supplementary Figure S1 for plasmid maps).

### Preparation of Cell Extracts

Cell growth, collection, and extracts were prepared as described previously (Liu et al., 2020). In brief, *E. coli* BL21 Star (DE3) cells were grown in 1L of 2xYTPG media, induced with 1 mM IPTG (OD_600_ = 0.6–0.8) to express T7 RNA polymerase, and harvested by centrifugation at an OD_600_ of around 3.0. Then, cell pellets were washed three times with cold S30 Buffer (10 mM Tris-acetate, 14 mM magnesium acetate, and 60 mM potassium acetate). After the final wash and centrifugation, the pelleted cells were resuspended in S30 Buffer (1 ml per Gram wet cell mass) and lysed by sonication (10 s on/off, 50% of amplitude, input energy ∼600 J) on ice. The lysate was then centrifuged twice at 12,000 g and 4°C for 10 min. The resulting supernatant (i.e., cell extracts) was flash frozen in liquid nitrogen and stored at −80°C until use.

### Cell-free Protein Synthesis Reactions

Standard CFPS reactions were carried out in 1.5-ml microcentrifuge tubes. Each reaction (15 μL) contains the following components: 12 mM magnesium glutamate, 10 mM ammonium glutamate, 130 mM potassium glutamate, 1.2 mM ATP, 0.85 mM each of GTP, UTP, and CTP, 34 μg/ml folinic acid, 170 μg/ml of *E. coli* tRNA mixture, 2 mM each of 20 standard amino acids, 0.33 mM nicotinamide adenine dinucleotide (NAD), 0.27 mM coenzyme A (CoA), 1.5 mM spermidine, 1 mM putrescine, 4 mM sodium oxalate, 33 mM phosphoenolpyruvate (PEP), 13.3 μg/ml plasmid unless otherwise noted, and 27% (v/v) of cell extracts. T7 RNA polymerase was not added to CFPS reactions since it was induced with IPTG during cell growth and contained in the cell extracts. CFPS expressed proteins were analyzed by SDS-PAGE and Western-blot.

### Modular Cell-free Protein Synthesis Reactions

To assemble enzymatic pathways for the synthesis of (*S*)-PED and 2-PE, one upstream module (module 1) and two downstream modules (module 2 and module 3) were constructed in cell-free systems. Each module of the CFPS reaction was performed at 30°C with a total volume of 15 μL in the 1.5-ml tube. In the first module, three enzymes were coexpressed by adding 13.3 μg/ml of pET28a-PAL2, 13.3 μg/ml of pET28a-Fdc1, and 6.7 μg/ml of pET28a-Pad1, respectively. This upstream CFPS module was incubated for 2 h to express enzymes for the conversion of L-Phe to styrene. In module 2, StyA, StyB, and SpEH were coexpressed with plasmid concentrations of 13.3, 3.3, and 13.3 μg/ml, respectively, for 6 h before mixing with module 1 to convert styrene to (*S*)-PED. Similarly, in module 3, StyA and StyB were expressed the same as in module 2; StyC was expressed with 13.3 μg/ml of pET28a-StyC. Coexpression of three enzymes in module 3 was also carried out for 6 h before mixing with module 1. Cell-free expressed StyA, StyB, and StyC together with endogenous ADHs can convert styrene to 2-PE. After mixing module 1 with module 2 or 3, 1 mM of the substrate L-Phe was added to the CFPS mixture and the reaction was further carried out for 16 h at 30°C to synthesize target compounds.

### Analytical Methods

All intermediates and final products were extracted with 2 volume of ethyl acetate for twice (as thoroughly as possible), followed by analysis with GC-MS (Trace 1300-ISQ, ThermoFisher Scientific) using a TG-5MS (30 m × 0.25 mm x 0.25 μm) column. For (*S*)-PED and 2-PE analysis, 1 μL of each sample was injected with a split ratio of 33.3:1. Helium was used as the carrier gas at a flow rate of 1.2 ml/min. The initial column temperature was 40°C and then increased at 30°C/min to 270°C (for (*S*)-PED) or 200°C (for 2-PE), which was maintained for 3 min. Afterwards, the column temperature increased at 30°C/min to 300°C with a final 5 min hold. The retention times of (*S*)-PED and 2-PE were 6.04 and 5.07 min, respectively. The concentration of each product was determined by comparison to a linear standard curve generated with a commercial standard (Supplementary Figure S2). All measurements were performed in triplicate.

## Results and Discussion

### Cell-free Expression of Each Enzyme and Demonstration of Their Catalytic Activities

To synthesize (*S*)-PED and 2-PE from the same precursor L-Phe, we chose in total seven enzymes from different organism sources to construct two full enzymatic pathways (Figure 1B). First, L-Phe is converted to styrene, which is a shared upstream intermediate, by successive deamination and decarboxylation with three enzymes PAL2, Fdc1, and Pad1 (Cochrane et al., 2004; Payne et al., 2015). Then, styrene undergoes two divergent pathways to form (*S*)-PED and 2-PE, respectively, with enzymes StyA, StyB, StyC, SpEH, and *E. coli* endogenous ADHs (Panke et al., 1998; Wu et al., 2013). Prior to build *in vitro* full pathways, each enzyme except the endogenous ADHs has to be actively expressed in CFPS reactions. To this end, the well-developed *E. coli*-based CFPS system was used to express all enzymes and test their catalytic activities. Note that the conversion of cinnamic acid to styrene requires two enzymes Fdc1 and Pad1 originated from *A. niger* (Payne et al., 2015); in addition, the styrene monooxygenase (SMO) from *Pseudomonas* sp. VLB120 also consists of two subunit enzymes StyA and StyB to oxidize styrene to styrene oxide (Panke et al., 1998). Therefore, these two enzymatic conversion steps need the coexpression of two related enzymes in one-pot CFPS reactions.

**FIGURE 2:**
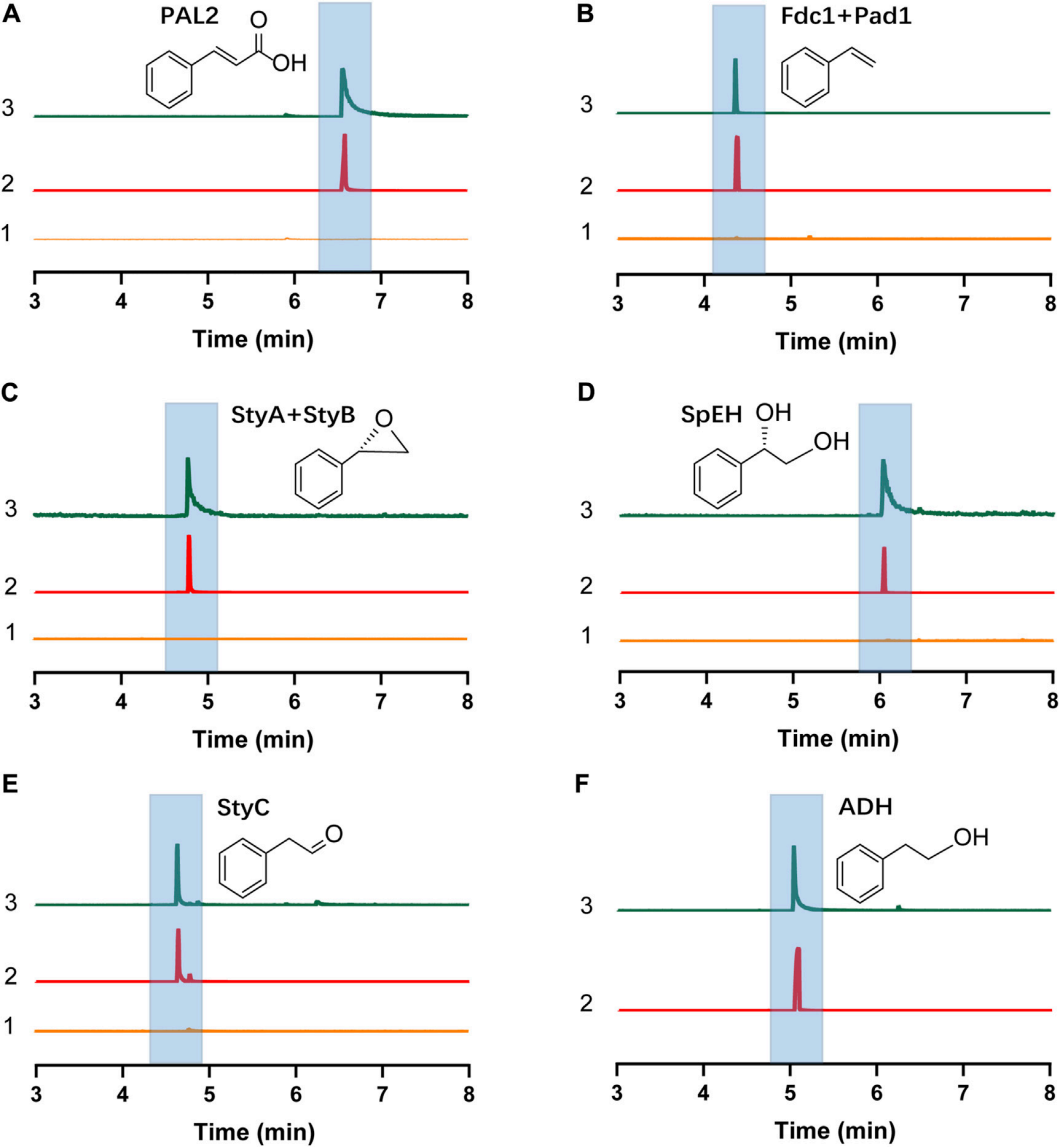
GC-MS detection of the product of each step enzymatic reaction. **(A)** Cinnamic acid. **(B)** Styrene. **(C)** Styrene oxide. **(D)** *(S)*-PED. **(E)** Phenylacetaldehyde. **(F)** 2-PE. In each panel, the numbers 1, 2, and 3 represent negative control without plasmid in the CFPS reaction (substrate was added), chemical standard, and cell-free reaction sample, respectively. Note that in **(F)**, no exogenous enzymes were expressed in CFPS and only *E. coli* endogenous ADHs catalyzed the reaction.

The *E. coli* CFPS system has been used to express various proteins, for example, therapeutic proteins, membrane proteins, and non-natural amino acid modified proteins (Henrich et al., 2015; Martin et al., 2018; Wilding et al., 2019), demonstrating the robustness of *E. coli* CFPS for *in vitro* protein production. To see if all enzymes we selected can be expressed, we used the *E. coli* strain BL21 Star (DE3) to prepare cell extracts for protein expression. We observed that each enzyme was successfully expressed in CFPS as shown by the Western-blot analysis (Supplementary Figure S3). Then, the catalytic ability of each enzyme was investigated. To do this, the substrate of each enzyme was added to the reaction mixture when the enzyme expression started. As shown in Figures 2A–E, each target compound was detected by GC-MS, demonstrating that all cell-free expressed enzymes were active to convert their substrates to the corresponding products (see Supplementary Figure S4 for MS spectra of all compounds). In addition, *E. coli* endogenous ADHs were also active to reduce phenylacetaldehyde to 2-PE when no exogenous enzymes were expressed by CFPS (Figure 2F). Taken together, the results demonstrated the potential of using CFPS to express functional enzymes to reconstitute (*S*)-PED and 2-PE biosynthetic pathways *in vitro*. As the goal of this work was to establish modular cell-free systems for tunable biotransformation, we next sought to coexpress different sets of enzymes in different single pot CFPS reactions and then mix them for synthesizing final products (Figure 1C).

### Designing Modular Cell-free Systems for the Synthesis of (S)-PED and 2-PE

**FIGURE 3:**
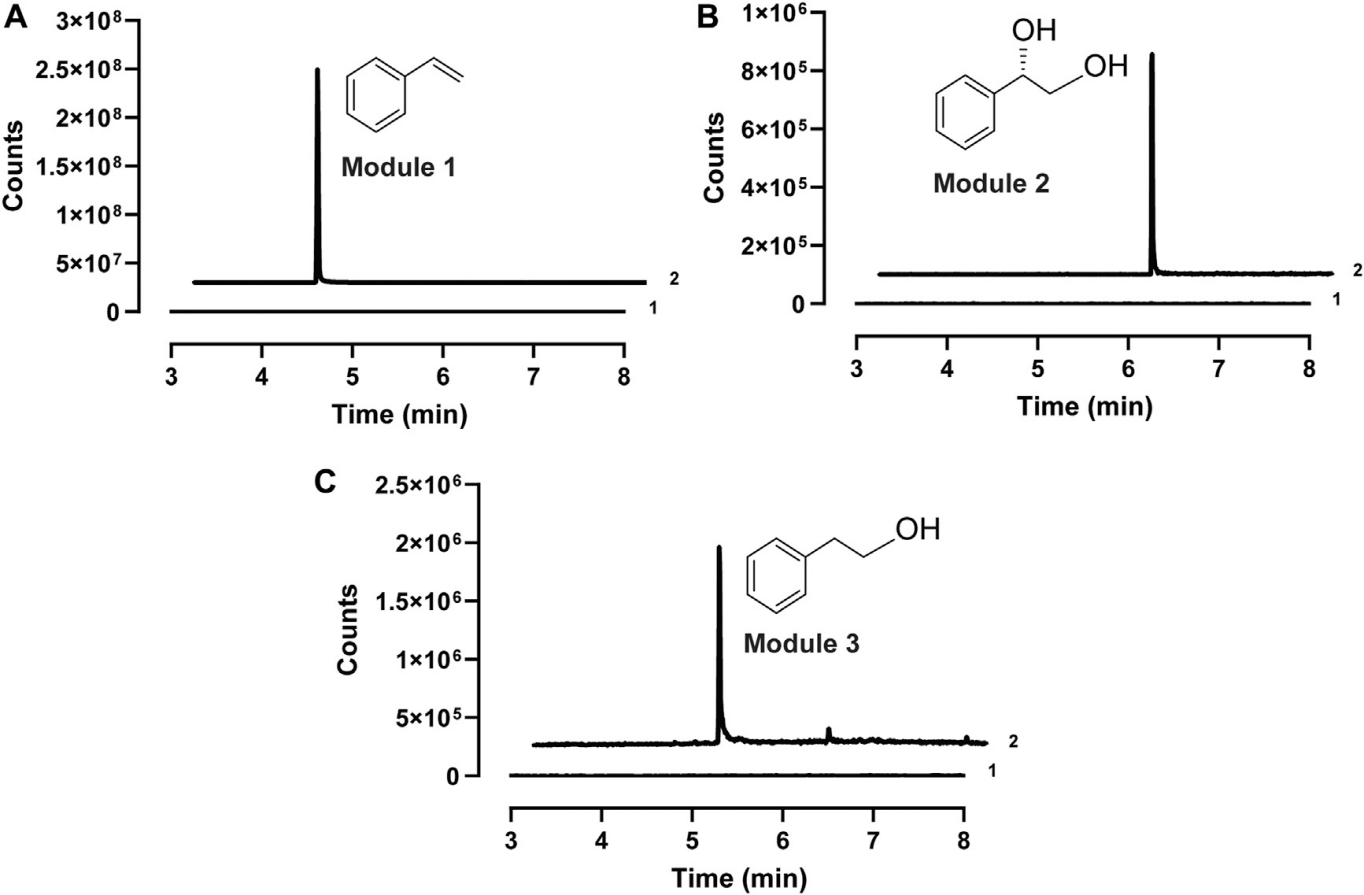
Extracted ion chromatograms (EIC) of the GC-MS detection of **(A)** styrene (m/z 140) in module 1, **(B)** (*S*)-PED (m/z 138) in module 2, and **(C)** 2-PE (m/z 122) in module 3. In each panel, 1, sample at the reaction time of zero; 2, sample from the reaction finished.

After demonstrating the enzyme activity, we next aimed to design CFPS modules for the expression of different enzyme combinations. The key idea was to express short pathways first and then mix them together to assemble the full pathways. Since styrene is the same upstream intermediate of (*S*)-PED and 2-PE, three enzymes (i.e., PAL2, Fdc1, and Pad1) were coexpressed in the first module of CFPS to convert L-Phe to styrene, which can be used by the downstream modules as a substrate. In module 2, styrene undergoes epoxidation and hydrolysis by SMO (i.e., StyA and StyB) and SpEH, respectively, to form (*S*)-PED. While cell-free expressed StyA, StyB, and StyC in module 3 catalyze the cascade conversion of styrene to phenylacetaldehyde, which can be reduced to 2-PE by endogenous ADHs. Initially, cell-free synthesized enzymes in each module were confirmed by Western-blot (Supplementary Figure S5). Next, biotransformations in all three modules were tested individually by adding 1 mM of substrate to the CFPS reactions. After the reaction, each product was extracted from the reaction mixture and analyzed by GC-MS. The results showed that all three target compounds of styrene, (*S*)-PED, and 2-PE were detected from the related modules (Figure 3), suggesting that our designed cell-free modules are capable of assembling long, full pathways for tunable biotransformation.

We, therefore, set out to build cell-free pathway combinations for (*S*)-PED and 2-PE synthesis. To achieve this goal, we first ran three cell-free modular reactions separately at 30°C to express enzymes and then mixed module 1 with module 2 or 3. While the time for enzyme expression in modules 2 and 3 was 6 h, the upstream module 1 was only incubated for 2 h before mixing because we observed that 2-h CFPS reaction was the best for module 1 to convert L-Phe to styrene (Supplementary Figure S6). When mixing two modules, 1 mM of L-Phe was added to the cell-free mixture and the reaction was further carried out for 16 h at 30°C to synthesize (*S*)-PED and 2-PE. The GC-MS analyses suggested that in both cases the final products were successfully detected (Supplementary Figure S7), demonstrating the ability of using modular cell-free reactions each with several CFPS-expressed enzymes to assemble long metabolic pathways *in vitro*.

### Effect of L-Phe on the Synthesis of (S)-PED and 2-PE

**FIGURE 4:**
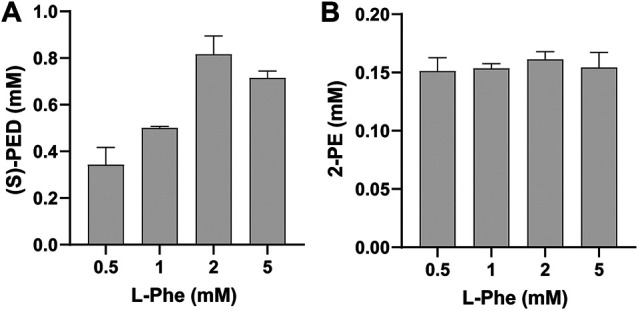
Effect of L-Phe on the synthesis of **(A)** (*S*)-PED and **(B)** 2-PE. Values show means with error bars representing standard deviations (s.d.) of at least three independent experiments.

Having validated the ability to mix cell-free modules for biotransformation, we next wanted to investigate the effect of L-Phe on the synthesis of (*S*)-PED and 2-PE, respectively. To do so, we mixed module 1 with module 2 or 3 after cell-free enzyme expression and added L-Phe to the reaction mixture at different final concentrations from 0.5 to 5 mM. Our data indicated that the effect of L-Phe on the synthesis of (*S*)-PED was more obvious than that on the synthesis of 2-PE (Figure 4). The highest titer of (*S*)-PED reached 0.82 mM when L-Phe was supplied with 2 mM, which is 2.4-fold higher than the titer achieved with 0.5 mM L-Phe (Figure 4A). By contrast, the titers of 2-PE were similar with different L-Phe concentrations, although 2 mM L-Phe gave rise to a bit higher titer of 2-PE as compared to all other concentrations (Figure 4B). In order to further optimize our system for enhanced production, we chose to add 2 mM of L-Phe to the reactions in our following experiments.

### Optimization of Reaction Temperature and Volume on the Synthesis of (S)-PED and 2-PE

**FIGURE 5:**
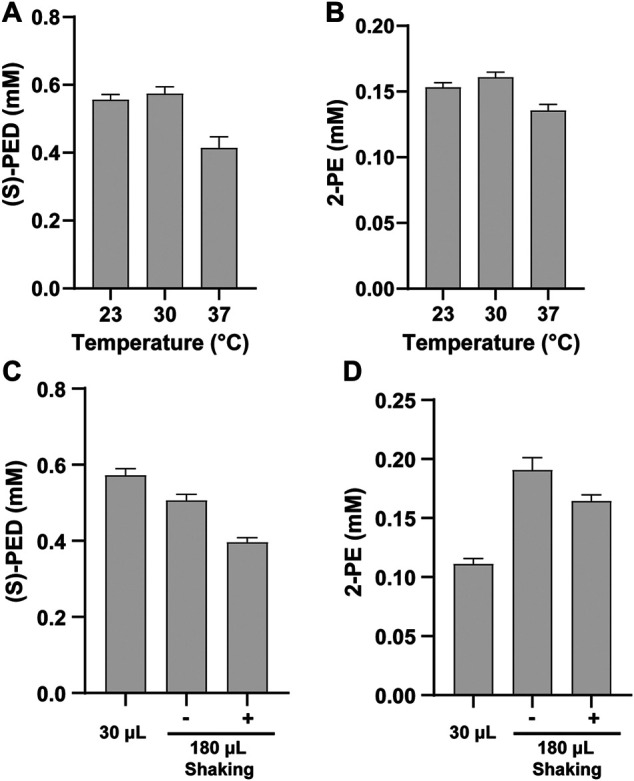
Effects of **(A**, **B)** reaction temperature and **(C**, **D)** reaction volume on the synthesis of (*S*)-PED and 2-PE, respectively. Values show means with error bars representing standard deviations (s.d.) of at least three independent experiments.

Cell-free reaction temperature is an important factor for optimization because it affects protein expression and enzyme activity. Our cell-free biosynthesis system includes two phases: the initial CFPS reaction phase and the second enzymatic biotransformation phase. While the best temperature for protein expression in *E. coli* CFPS system has been found to be 30°C (Goering et al., 2017; Li et al., 2016; Martin et al., 2018), whether this temperature is optimum for the enzymatic reactions remaining unknown. Therefore, we did not focus on the optimization of the CFPS temperature in this work. Instead, we conducted our optimization by investigating the optimal reaction temperature during the biotransformation stage for producing (*S*)-PED and 2-PE. After mixing two cell-free modules, we incubated the reactions containing 2 mM of L-Phe at 23, 30, and 37°C. The results indicated that low temperatures favored the product formation and the maximum titers of both products were obtained at 30°C (Figures 5A,B). This makes the whole production process easy without the change of incubation temperature from protein expression to product synthesis. Our finding is similar to previous reports that 30°C is the optimal temperature to synthesize enzymes and the resulting chemical compounds (Goering et al., 2017; Grubbe et al., 2020; Liu et al., 2020).

Next, we evaluated the effect of reaction volume on the synthesis of (*S*)-PED and 2-PE. We ran standard CFPS reactions in 15 μL volume and also scaled up the volume to 90 μL per reaction. Thus, the total volume after mixing two modules was 30 μL or 180 μL. For the group of reaction volume with 180 μL, we also compared the reactions without shaking and with shaking at 600 rpm. All enzymatic reactions were carried out at 30°C for 16 h and the final products were extracted and analyzed. Interestingly, we found that the reaction volume has different effects on the product titers (Figures 5C,D). For (*S*)-PED, the titer obtained from the reaction volume of 30 μL was higher than that of the volume of 180 μL. By contrast, the titer of 2-PE in the reaction volume of 180 μL was maximum, but shaking was not necessary. In both cases, the product titers in 180 μL volume with shaking were lower than those without shaking. While the impact of reaction volume on product formation was different, our results suggested that 1) the reaction volume should be investigated for each new enzymatic pathway *in vitro* and 2) cell-free system is flexible for manipulation and optimization. With the aim to improve each product titer, we decided to use 30 and 180 μL reaction volumes to synthesize (*S*)-PED and 2-PE, respectively, in the following studies.

### Enhancing the Epoxidation Step of Styrene for Efficient Biotransformation

Styrene accumulated in the first cell-free module is the upstream precursor for the synthesis of (*S*)-PED in module 2 and 2-PE in module 3. It is, therefore, important to efficiently convert the water-insoluble styrene to styrene oxide, which can be used by the subsequent enzymes for further conversion. The epoxidation of styrene is catalyzed by the enzyme SMO (i.e., StyA and StyB). Previous studies have shown that StyA plays a core role in the biocatalytic activity of SMO and StyB helps maximize the epoxidation of styrene (Panke et al., 1998; Otto et al., 2004). In addition, the highest epoxidation activity can be achieved when the molar ratio of StyA and StyB reaches at about 1:1 (Otto et al., 2004; Corrado et al., 2018). Based on these reports, we were curious to know if tuning StyA and StyB might enhance the epoxidation activity of styrene. Thus, we subsequently sought to test two strategies to see their effects on the efficiency of cell-free biotransformations. First, we overexpressed the key enzyme StyA *in vivo* in *E. coli* rather than in CFPS and prepared StyA-enriched cell extracts to coexpress the rest enzymes, for example, StyB, StyC, and SpEH, in CFPS reactions. Second, we fused StyA and StyB with a flexible linker to construct a fused SMO (StyA-linker-StyB) and, as a result, the molar ratio of the two enzymes should be 1:1 once they are expressed in CFPS.

**FIGURE 6:**
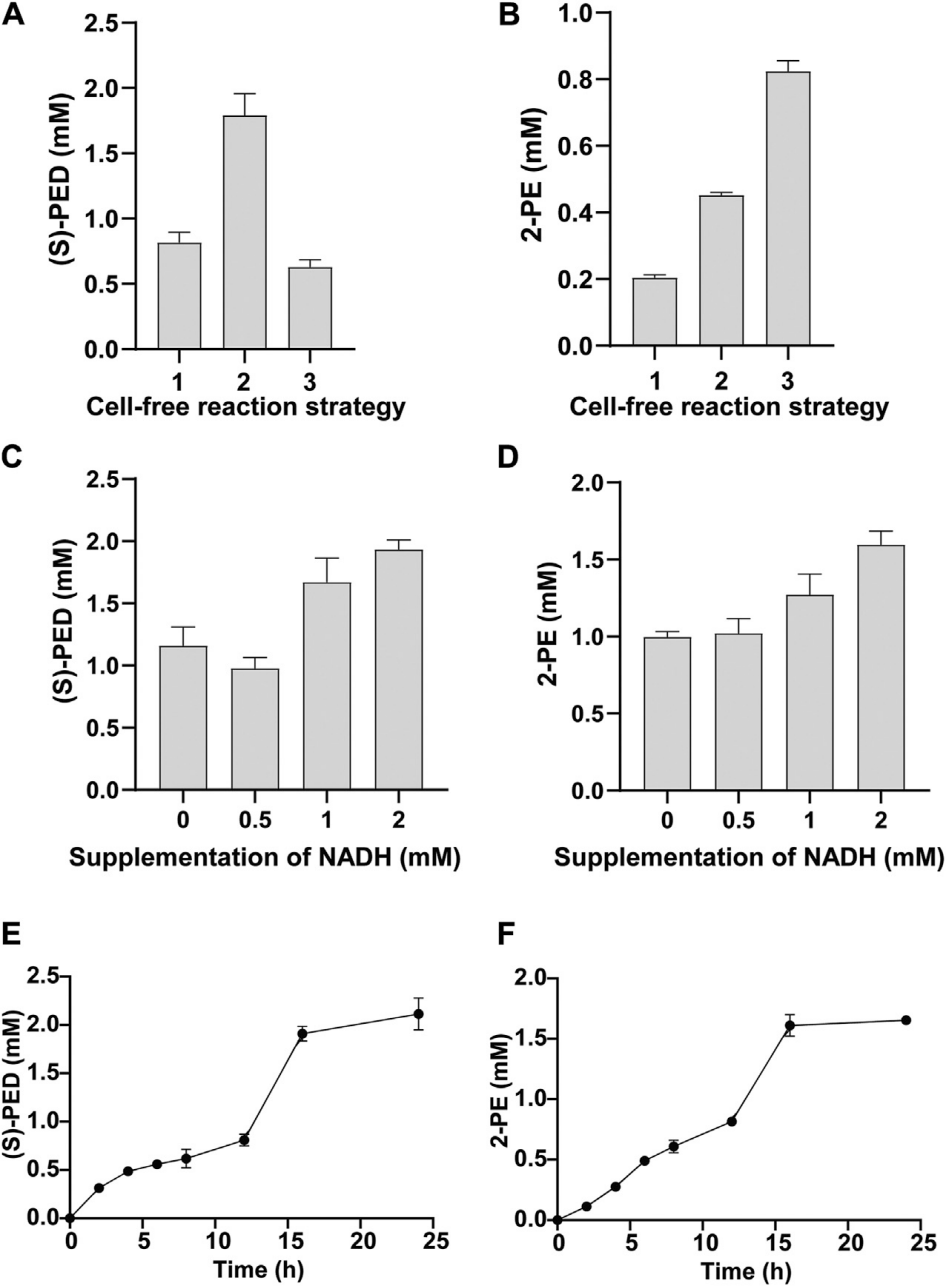
Optimization of StyA and StyB expression for the synthesis of **(A)** (*S*)-PED and **(B)** 2-PE. In panels **(A)** and **(B)**: 1, both StyA and StyB were expressed in CFPS; 2, StyA was expressed *in vivo* to prepare cell extracts and StyA-enriched CFPS was used to express StyB; 3, StyA and StyB was fused and the resulting StyA-linker-StyB was expressed in CFPS. Effect of supplementation of NADH on **(C)** (*S*)-PED and **(D)** 2-PE formation. Time courses of **(E)** (*S*)-PED and **(F)** 2-PE production within 24 h. Values show means with error bars representing standard deviations (s.d.) of at least three independent experiments.

By using the above-mentioned two strategies, we found that they did impact the final product titers, albeit their effects were different. For the synthesis of (*S*)-PED, StyA-enriched CFPS system enabled the maximum conversion of styrene to (*S*)-PED, giving rise to the titer of 1.79 ± 0.17 mM (Figure 6A), which is > 2 times higher than the control reaction by coexpression of StyA and StyB with plasmids. However, cell-free expression of fused SMO (StyA-linker-StyB) led to the lowest production of (*S*)-PED. The results are in agreement with our previous report that cell extracts pre-enriched with StyA notably enhanced the cascade biotransformation of styrene to (*S*)-PED (Liu et al., 2020). In contrast, both StyA-enriched CFPS and fused SMO benefited the 2-PE synthesis and significantly improved the titers, which are >2 and >4 times higher than that of the control reaction (0.20 ± 0.01 mM) (Figure 6B). Interestingly, not like (*S*)-PED, fused SMO was better than the StyA-enriched CFPS to produce 2-PE, perhaps as a result of the different downstream enzymes (SpEH and StyC/ADHs in (*S*)-PED and 2-PE pathways, respectively) that influence the whole cell-free metabolic fluxes.

Since the activity of StyB is dependent on NADH as an electron donor (Otto et al., 2004), we next wanted to supplement NADH to the cell-free systems and explore the effect of NADH concentration on product formation. As shown in Figures 6C,D, the titers of both products were obviously increased by supplementation of NADH. Specifically, 1.93 ± 0.08 mM of (*S*)-PED (96.5% conversion based on 2 mM L-Phe) and 1.60 ± 0.09 mM of 2-PE (80% conversion based on 2 mM L-Phe) were produced by adding 2 mM of NADH per reaction. Notably, we also observed product formation without NADH supplementation. This is because our cell-free system, which is based on the *E. coli* crude lysate, contains endogenous NADH that can be used to support the activity of StyB. Additionally, in CFPS reactions 0.33 mM of NAD^+^ was added that can also be reduced to NADH by the native NADH/NAD^+^ recycling systems (Jewett et al., 2008).

Finally, with the optimized cell-free reaction system, we tested the time courses of product formation by measuring (*S*)-PED and 2-PE concentrations at different time points (Figures 6E,F). After mixing two cell-free modules, the cascade biotransformation was carried out for 24 h in total. Our data indicated that the biosynthesis of (*S*)-PED occurred with a gradual increase before 12 h, while 2-PE underwent a nearly linear increase manner. Clearly, more than 95% of both products were synthesized during the initial 16 h reaction and no obvious products were accumulated from 16 to 24 h. Eventually, the conversion rates based on the starting substrate (2 mM L-Phe) at 24 h were 100 and 82.5% for (*S*)-PED and 2-PE, respectively. Since styrene is the common upstream intermediate, the standard Gibbs free energy change of the overall reaction from styrene to (*S*)-PED and 2-PE were calculated to be −363.92 kJ/mol and −224.66 kJ/mol (https://www.chemeo.com/), respectively. This demonstrates that the conversion of styrene to (*S*)-PED is more thermodynamically favorable than that to 2-PE, which is in agreement with the experimental data.

In the present work, we demonstrated the robustness and feasibility of using the *E. coli*-based CFPS system to assemble long biosynthetic pathways enabled by designing modular cell-free systems. While the two paradigms were successful, the overall conversion rates from L-Phe to (*S*)-PED and 2-PE were different. One main reason might be the different levels of the concerted activity of each pathway reconstituted with multiple cell-free expressed enzymes. For example, the conversion of L-Phe to 2-PE was not complete within 24 h, which is likely due to the low expression of soluble StyC and the resulting low catalytic efficiency. Therefore, given the flexibility of cell-free systems, one might switch from the *E. coli* CFPS system to many other CFPS systems (Kelwick et al., 2016; Li et al., 2017; Des Soye et al., 2018; Wang et al., 2018; Xu et al., 2020; Zhang et al., 2020) to better mimic the physicochemical environment of native hosts for the expression of related enzymes with high (soluble) yields and high activities. In other words, various CFPS systems can be used to express highly active enzymes in different cell-free modules and then mix them for efficient biotranformations, thus giving rise to high productivity.

## Conclusion

In this study, we demonstrated the design of modular cell-free systems to build *in vitro* long metabolic pathways for tunable conversion of L-Phe to two aromatic compounds (*S*)-PED and 2-PE. The activities of all cell-free expressed enzymes were first confirmed and then mixed modules were able to synthesize (*S*)-PED or 2-PE. After optimization, the final conversion rates based on L-Phe (2 mM) at 24 h were 100% for (*S*)-PED and 82.5% for 2-PE. Looking forward, we envision that modular cell-free systems will provide a feasible approach to construct long enzymatic pathways for the synthesis of valuable chemicals of pharmaceutical and industrial importance.

## Data Availability

The original contributions presented in the study are included in the article/Supplementary Material, further inquiries can be directed to the corresponding author.

## References

[B1] Bornscheuer U. T. Huisman G. W. Kazlauskas R. J. Lutz S. Moore J. C. Robins K. (2012). Engineering the Third Wave of Biocatalysis. Nature 485, 185–194. 10.1038/nature11117 22575958

[B2] Bowie J. U. Sherkhanov S. Korman T. P. Valliere M. A. Opgenorth P. H. Liu H. (2020). Synthetic Biochemistry: The Bio-Inspired Cell-free Approach to Commodity Chemical Production. Trends Biotechnol. 38, 766–778. 10.1016/j.tibtech.2019.12.024 31983463

[B3] Bundy B. C. Hunt J. P. Jewett M. C. Swartz J. R. Wood D. W. Frey D. D. (2018). Cell-free Biomanufacturing. Curr. Opin. Chem. Eng. 22, 177–183. 10.1016/j.coche.2018.10.003

[B4] Cochrane F. C. Davin L. B. Lewis N. G. (2004). The *Arabidopsis* Phenylalanine Ammonia Lyase Gene Family: Kinetic Characterization of the Four PAL Isoforms. Phytochemistry 65, 1557–1564. 10.1016/j.phytochem.2004.05.006 15276452

[B5] Corrado M. L. Knaus T. Mutti F. G. (2018). A Chimeric Styrene Monooxygenase with Increased Efficiency in Asymmetric Biocatalytic Epoxidation. ChemBioChem 19, 679–686. 10.1002/cbic.201700653 29378090 PMC5900736

[B6] Cotton C. A. Claassens N. J. Benito-Vaquerizo S. Bar-Even A. (2020). Renewable Methanol and Formate as Microbial Feedstocks. Curr. Opin. Biotechnol. 62, 168–180. 10.1016/j.copbio.2019.10.002 31733545

[B7] Des Soye B. J. Davidson S. R. Weinstock M. T. Gibson D. G. Jewett M. C. (2018). Establishing a High-Yielding Cell-free Protein Synthesis Platform Derived fromVibrio Natriegens. ACS Synth. Biol. 7, 2245–2255. 10.1021/acssynbio.8b00252 30107122

[B8] Dudley Q. M. Karim A. S. Nash C. J. Jewett M. C. (2020). *In Vitro* prototyping of Limonene Biosynthesis Using Cell-free Protein Synthesis. Metab. Eng. 61, 251–260. 10.1016/j.ymben.2020.05.006 32464283

[B9] Feng J. Yang C. Zhao Z. Xu J. Li J. Li P. (2021). Application of Cell-free Protein Synthesis System for the Biosynthesis of L-Theanine. ACS Synth. Biol. 10, 620–631. 10.1021/acssynbio.0c00618 33719397

[B10] Goering A. W. Li J. McClure R. A. Thomson R. J. Jewett M. C. Kelleher N. L. (2017). *In Vitro* reconstruction of Nonribosomal Peptide Biosynthesis Directly from DNA Using Cell-free Protein Synthesis. ACS Synth. Biol. 6, 39–44. 10.1021/acssynbio.6b00160 27478992 PMC5250536

[B11] Grubbe W. S. Rasor B. J. Krüger A. Jewett M. C. Karim A. S. (2020). Cell-free Styrene Biosynthesis at High Titers. Metab. Eng. 61, 89–95. 10.1016/j.ymben.2020.05.009 32502620

[B12] Henrich E. Hein C. Dötsch V. Bernhard F. (2015). Membrane Protein Production inEscherichia Colicell-free Lysates. FEBS Lett. 589, 1713–1722. 10.1016/j.febslet.2015.04.045 25937121

[B13] Humphreys C. M. Minton N. P. (2018). Advances in Metabolic Engineering in the Microbial Production of Fuels and Chemicals from C1 Gas. Curr. Opin. Biotechnol. 50, 174–181. 10.1016/j.copbio.2017.12.023 29414057

[B14] Jewett M. C. Calhoun K. A. Voloshin A. Wuu J. J. Swartz J. R. (2008). An Integrated Cell‐free Metabolic Platform for Protein Production and Synthetic Biology. Mol. Syst. Biol. 4, 220. 10.1038/msb.2008.57 18854819 PMC2583083

[B15] Karim A. S. Dudley Q. M. Juminaga A. Yuan Y. Crowe S. A. Heggestad J. T. (2020). *In Vitro* prototyping and Rapid Optimization of Biosynthetic Enzymes for Cell Design. Nat. Chem. Biol. 16, 912–919. 10.1038/s41589-020-0559-0 32541965

[B16] Karim A. S. Jewett M. C. (2016). A Cell-free Framework for Rapid Biosynthetic Pathway Prototyping and Enzyme Discovery. Metab. Eng. 36, 116–126. 10.1016/j.ymben.2016.03.002 26996382

[B17] Kelwick R. Ricci L. Chee S. M. Bell D. Webb A. J. Freemont P. S. (2018). Cell-free Prototyping Strategies for Enhancing the Sustainable Production of Polyhydroxyalkanoates Bioplastics. Synth. Biol. 3, ysy016. 10.1093/synbio/ysy016 PMC7445755 32995523

[B18] Kelwick R. Webb A. J. MacDonald J. T. Freemont P. S. (2016). Development of a *Bacillus Subtilis* Cell-free Transcription-Translation System for Prototyping Regulatory Elements. Metab. Eng. 38, 370–381. 10.1016/j.ymben.2016.09.008 27697563

[B19] Ko Y.-S. Kim J. W. Lee J. A. Han T. Kim G. B. Park J. E. (2020). Tools and Strategies of Systems Metabolic Engineering for the Development of Microbial Cell Factories for Chemical Production. Chem. Soc. Rev. 49, 4615–4636. 10.1039/D0CS00155D 32567619

[B20] Kwon Y.-C. Oh I.-S. Lee N. Lee K.-H. Yoon Y. J. Lee E. Y. (2013). Integrating Cell-free Biosyntheses of Heme Prosthetic Group and Apoenzyme for the Synthesis of Functional P450 Monooxygenase. Biotechnol. Bioeng. 110, 1193–1200. 10.1002/bit.24785 23172243

[B21] Li J. Lawton T. J. Kostecki J. S. Nisthal A. Fang J. Mayo S. L. (2016). Cell‐free Protein Synthesis Enables High Yielding Synthesis of an Active Multicopper Oxidase. Biotechnol. J. 11, 212–218. 10.1002/biot.201500030 26356243

[B22] Li J. Neubauer P. (2014). *Escherichia coli* as a Cell Factory for Heterologous Production of Nonribosomal Peptides and Polyketides. New Biotechnol. 31, 579–585. 10.1016/j.nbt.2014.03.006 24704144

[B23] Li J. Wang H. Kwon Y.-C. Jewett M. C. (2017). Establishing a High Yieldingstreptomyces-Based Cell-free Protein Synthesis System. Biotechnol. Bioeng. 114, 1343–1353. 10.1002/bit.26253 28112394

[B24] Li J. Zhang L. Liu W. (2018). Cell-free Synthetic Biology for *In Vitro* Biosynthesis of Pharmaceutical Natural Products. Synth. Syst. Biotechnol. 3, 83–89. 10.1016/j.synbio.2018.02.002 29900420 PMC5995452

[B25] Liu W.-Q. Zhang L. Chen M. Li J. (2019). Cell-free Protein Synthesis: Recent Advances in Bacterial Extract Sources and Expanded Applications. Biochem. Eng. J. 141, 182–189. 10.1016/j.bej.2018.10.023

[B26] Liu W. Q. Wu C. Jewett M. C. Li J. (2020). Cell‐free Protein Synthesis Enables One‐pot cascade Biotransformation in an Aqueous‐organic Biphasic System. Biotechnol. Bioeng. 117, 4001–4008. 10.1002/bit.27541 32827317

[B27] Lukito B. R. Wu S. Saw H. J. J. Li Z. (2019). One-Pot Production of Natural 2-Phenylethanol fromL-Phenylalanine via Cascade Biotransformations. ChemCatChem 11, 831–840. 10.1002/cctc.201801613

[B28] Martin R. W. Des Soye B. J. Kwon Y.-C. Kay J. Davis R. G. Thomas P. M. (2018). Cell-free Protein Synthesis from Genomically Recoded Bacteria Enables Multisite Incorporation of Noncanonical Amino Acids. Nat. Commun. 9, 1203. 10.1038/s41467-018-03469-5 29572528 PMC5865108

[B29] Moore S. J. Lai H.-E. Chee S.-M. Toh M. Coode S. Chengan K. (2021). A *Streptomyces Venezuelae* Cell-free Toolkit for Synthetic Biology. ACS Synth. Biol. 10, 402–411. 10.1021/acssynbio.0c00581 33497199 PMC7901020

[B30] Nielsen J. Keasling J. D. (2016). Engineering Cellular Metabolism. Cell 164, 1185–1197. 10.1016/j.cell.2016.02.004 26967285

[B31] Otto K. Hofstetter K. Röthlisberger M. Witholt B. Schmid A. (2004). Biochemical Characterization of StyAB from *Pseudomonas* Sp. Strain VLB120 as a Two-Component Flavin-Diffusible Monooxygenase. J. Bacteriol. 186, 5292–5302. 10.1128/JB.186.16.5292-5302.2004 15292130 PMC490909

[B32] Panke S. Witholt B. Schmid A. Wubbolts M. G. (1998). Towards a Biocatalyst for (S)-Styrene Oxide Production: Characterization of the Styrene Degradation Pathway of Pseudomonas Sp. Strain VLB120. Appl. Environ. Microbiol. 64, 2032–2043. 10.1128/AEM.64.6.2032-2043.1998 9603811 PMC106275

[B33] Payne K. A. P. White M. D. Fisher K. Khara B. Bailey S. S. Parker D. (2015). New Cofactor Supports α,β-unsaturated Acid Decarboxylation via 1,3-dipolar Cycloaddition. Nature 522, 497–501. 10.1038/nature14560 26083754 PMC4988494

[B34] Peng F. Ou X. Y. Zhao Y. Zong M. H. Lou W. Y. (2019). Highly Selective Resolution of Racemic 1‐phenyl‐1,2‐ethanediol by a Novel Strain Kurthia Gibsonii SC 0312. Lett. Appl. Microbiol. 68, 446–454. 10.1111/lam.13123 30702764

[B35] Rasor B. J. Vögeli B. Landwehr G. M. Bogart J. W. Karim A. S. Jewett M. C. (2021). Toward Sustainable, Cell-free Biomanufacturing. Curr. Opin. Biotechnol. 69, 136–144. 10.1016/j.copbio.2020.12.012 33453438

[B36] Sheldon R. A. Woodley J. M. (2018). Role of Biocatalysis in Sustainable Chemistry. Chem. Rev. 118, 801–838. 10.1021/acs.chemrev.7b00203 28876904

[B37] Silverman A. D. Karim A. S. Jewett M. C. (2020). Cell-free Gene Expression: an Expanded Repertoire of Applications. Nat. Rev. Genet. 21, 151–170. 10.1038/s41576-019-0186-3 31780816

[B38] Swartz J. R. (2018). Expanding Biological Applications Using Cell-free Metabolic Engineering: An Overview. Metab. Eng. 50, 156–172. 10.1016/j.ymben.2018.09.011 30367967

[B39] Wang H. Li J. Jewett M. C. (2018). Development of a *Pseudomonas Putida* Cell-free Protein Synthesis Platform for Rapid Screening of Gene Regulatory Elements. Synth. Biol. 3, ysy003. 10.1093/synbio/ysy003 PMC7445763 32995512

[B40] Wang Y. Zhang H. Lu X. Zong H. Zhuge B. (2019). Advances in 2-phenylethanol Production from Engineered Microorganisms. Biotechnol. Adv. 37, 403–409. 10.1016/j.biotechadv.2019.02.005 30768954

[B41] Wang Z. Sundara Sekar B. Li Z. (2021). Recent Advances in Artificial Enzyme Cascades for the Production of Value-Added Chemicals. Bioresour. Tech. 323, 124551–551. 10.1016/j.biortech.2020.124551 33360113

[B42] Wilding K. M. Hunt J. P. Wilkerson J. W. Funk P. J. Swensen R. L. Carver W. C. (2019). Endotoxin-Free E. Coli- Based Cell-free Protein Synthesis: Pre-expression Endotoxin Removal Approaches for On-Demand Cancer Therapeutic Production. Biotechnol. J. 14, 1800271. 10.1002/biot.201800271 30024107

[B43] Wu G. Yan Q. Jones J. A. Tang Y. J. Fong S. S. Koffas M. A. G. (2016). Metabolic burden: Cornerstones in Synthetic Biology and Metabolic Engineering Applications. Trends Biotechnol. 34, 652–664. 10.1016/j.tibtech.2016.02.010 26996613

[B44] Wu S. Li A. Chin Y. S. Li Z. (2013). Enantioselective Hydrolysis of Racemic and Meso-Epoxides with Recombinant *Escherichia coli* Expressing Epoxide Hydrolase from *Sphingomonas* Sp. HXN-200: Preparation of Epoxides and Vicinal Diols in High *Ee* and High Concentration. ACS Catal. 3, 752–759. 10.1021/cs300804v

[B45] Wu S. Li Z. (2018). Whole-cell cascade Biotransformations for One-Pot Multistep Organic Synthesis. ChemCatChem 10, 2164–2178. 10.1002/cctc.201701669

[B46] Wu S. Snajdrova R. Moore J. C. Baldenius K. Bornscheuer U. T. (2021). Biocatalysis: Enzymatic Synthesis for Industrial Applications. Angew. Chem. Int. Ed. 60, 88–119. 10.1002/ange.202006648 PMC7818486 32558088

[B47] Xu H. Liu W.-Q. Li J. (2020). Translation Related Factors Improve the Productivity of a *Streptomyces*-Based Cell-free Protein Synthesis System. ACS Synth. Biol. 9, 1221–1224. 10.1021/acssynbio.0c00140 32330385

[B48] Zhang L. Liu W.-Q. Li J. (2020). Establishing a Eukaryotic *Pichia pastoris* Cell-free Protein Synthesis System. Front. Bioeng. Biotechnol. 8, 536. 10.3389/fbioe.2020.00536 32626695 PMC7314905

[B49] Zhuang L. Huang S. Liu W.-Q. Karim A. S. Jewett M. C. Li J. (2020). Total *In Vitro* Biosynthesis of the Nonribosomal Macrolactone Peptide Valinomycin. Metab. Eng. 60, 37–44. 10.1016/j.ymben.2020.03.009 32224263 PMC8190601

