# Supplementary material for: Designing Modular Cell-free Systems for Tunable Biotransformation of l-phenylalanine to Aromatic Compounds
Source: Front Bioeng Biotechnol. 2021 Jul 28;9:730663. doi: 10.3389/fbioe.2021.730663 (PMC8355704; doi:10.3389/fbioe.2021.730663)
Supplement: Supplementary file 1 [file DataSheet1.PDF]

Supplementary Material

Designing Modular Cell-Free Systems for Tunable Biotransformation of L-phenylalanine to Aromatic Compounds

Chen Yang[1,2,3], Yushi Liu[1], Wan-Qiu Liu[1], Changzhu Wu[4], Jian Li[1,*]

[1]School of Physical Science and Technology, ShanghaiTech University, Shanghai 201210, China

[2]Shanghai Advanced Research Institute, Chinese Academy of Sciences, Shanghai 201203, China

[3]University of Chinese Academy of Sciences, Beijing 100049, China

[4]Danish Institute for Advanced Study (DIAS) and Department of Physics, Chemistry and Pharmacy, University of Southern Denmark, Odense 5230, Denmark

*Correspondence:
Jian Li
lijian@shanghaitech.edu.cn

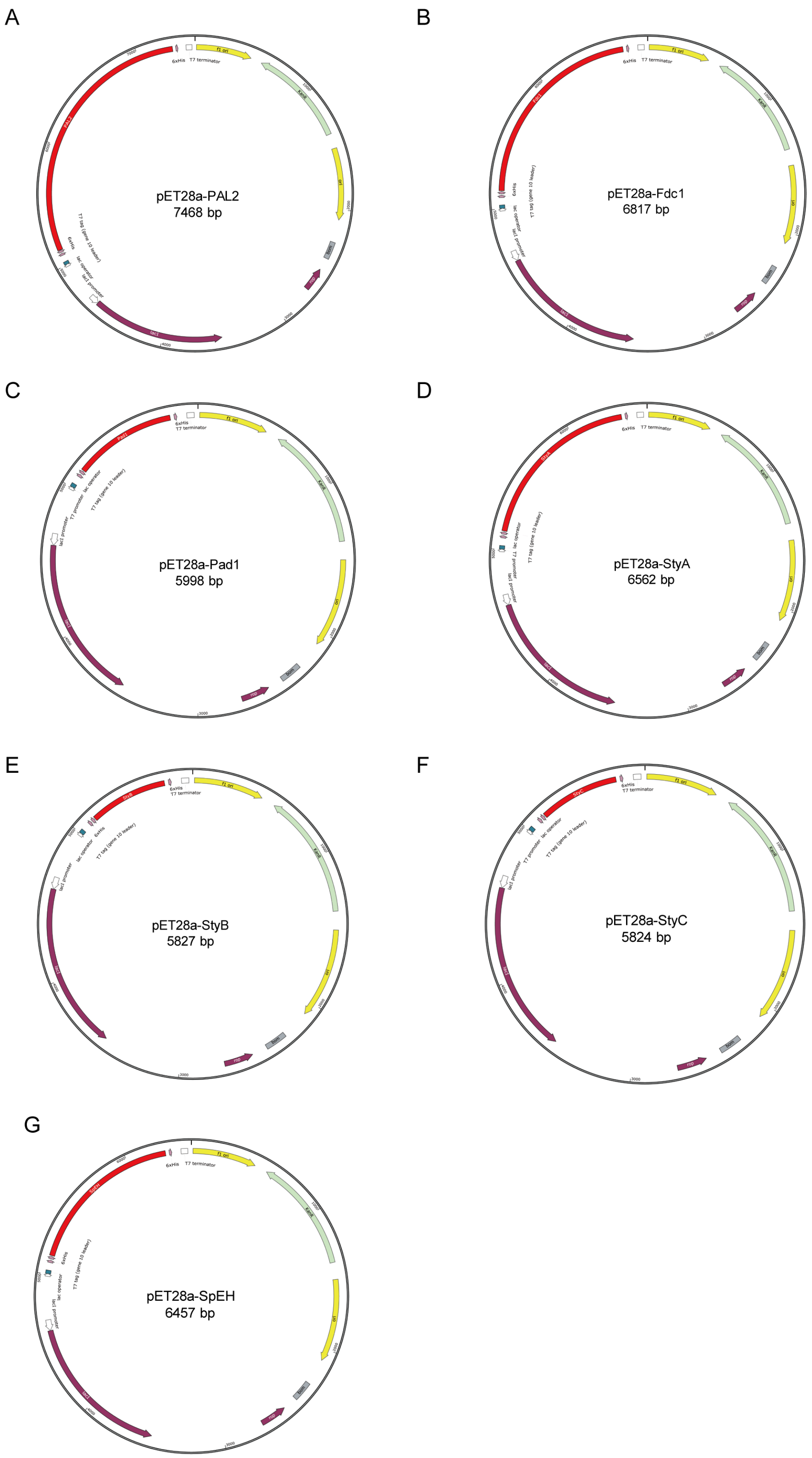

Figure S1. Plasmid maps of (A) pET28a-PAL2, (B) pET28a-Fdc1, (C) pET28a-Pad1, (D) pET28a-StyA, (E) pET28a-StyB, (F) pET28a-StyC, and (G) pET28a-SpEH.

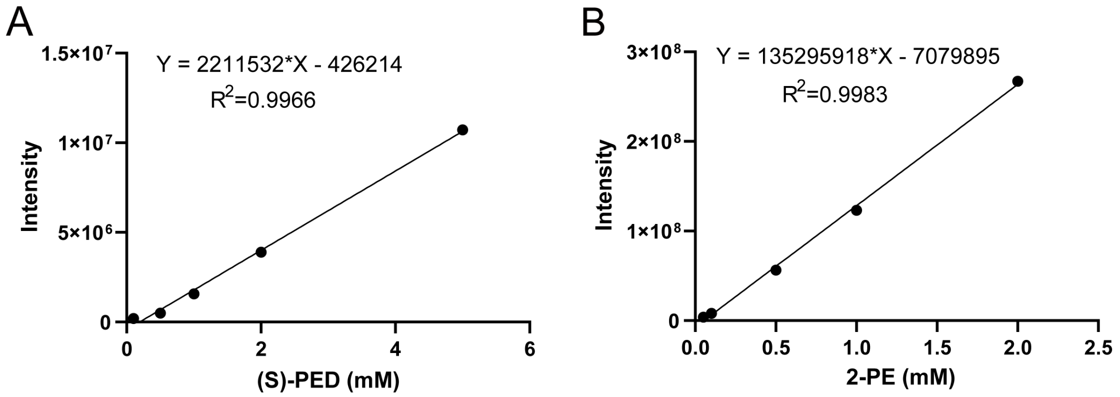

Figure S2. Standard curves of (A) (*S*)-PED and (B) 2-PE.

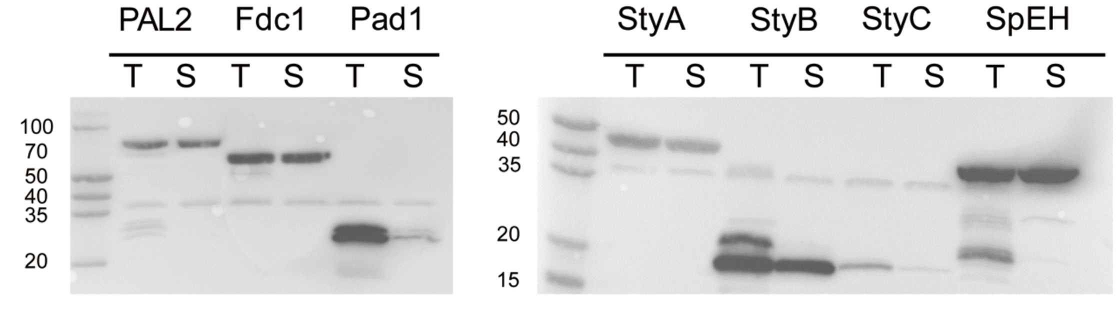

Figure S3. Western-blot analysis of individual expression of PAL2 (78.7 kDa), Fdc1 (55.2 kDa), Pad1 (21.6 kDa), StyA (46.3 kDa), StyB (18.4 kDa), StyC (18.0 kDa), and SpEH (42.9 kDa). T, total protein; S, soluble protein.

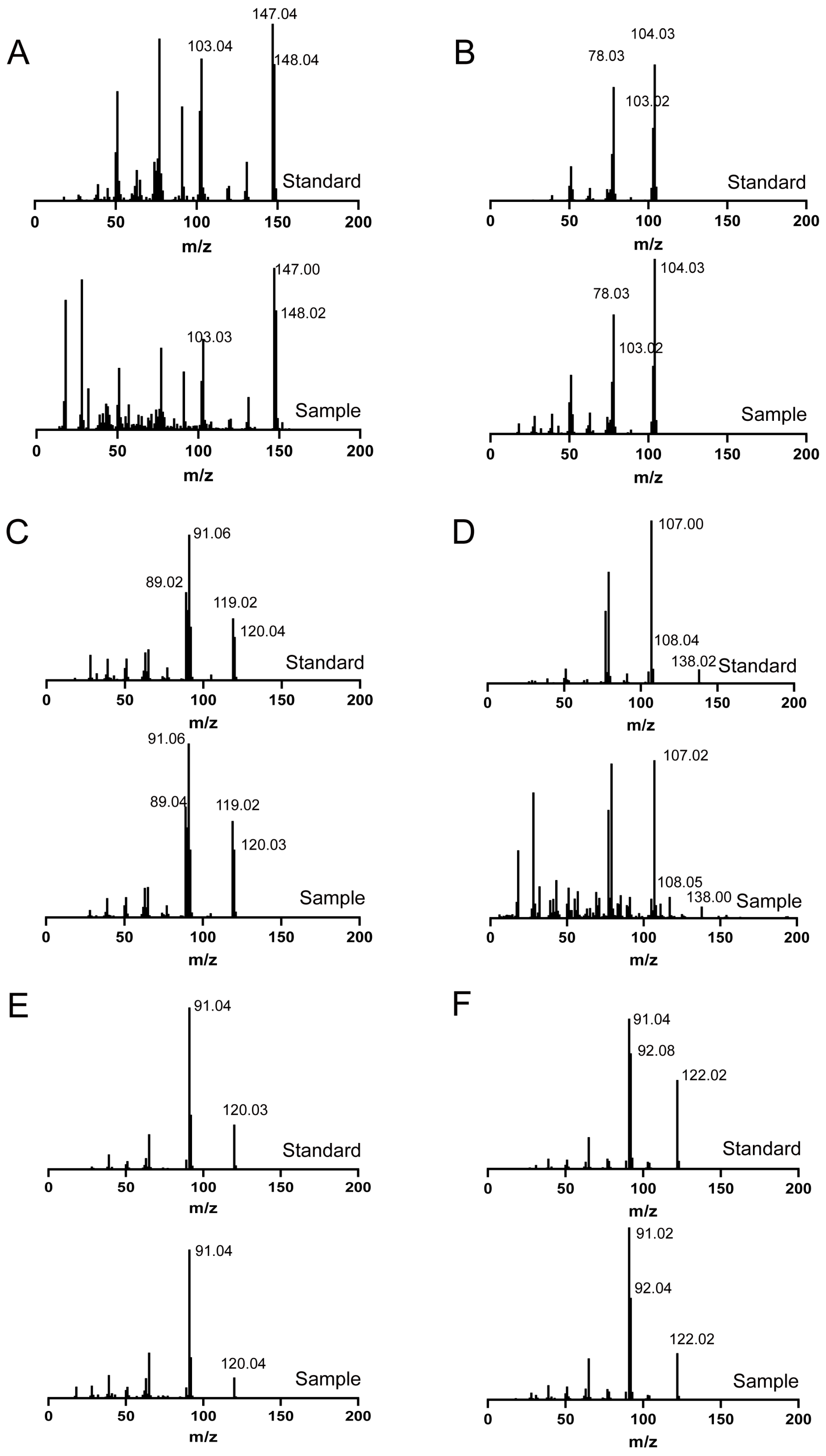

Figure S4. MS spectra of (A) cinnamic acid, (B) styrene, (C) styrene oxide, (D) (*S*)-PED, (E) phenylacetaldehyde, and (F) 2-PE. In each panel, top: standard compound; bottom: cell-free synthesized sample.

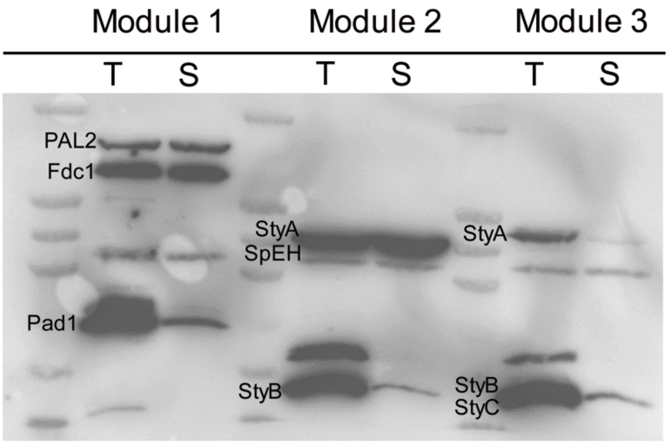

Figure S5. Western-blot analysis of coexpression of enzymes in three cell-free modules. T, total protein; S, soluble protein.

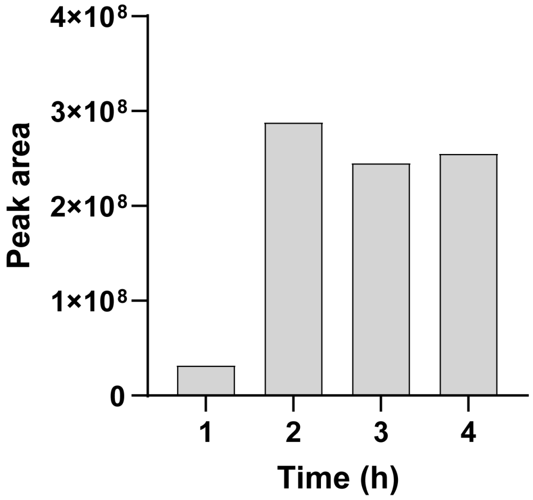

Figure S6. The time for adding L-Phe to the first cell-free module and its effect on the synthesis of styrene.

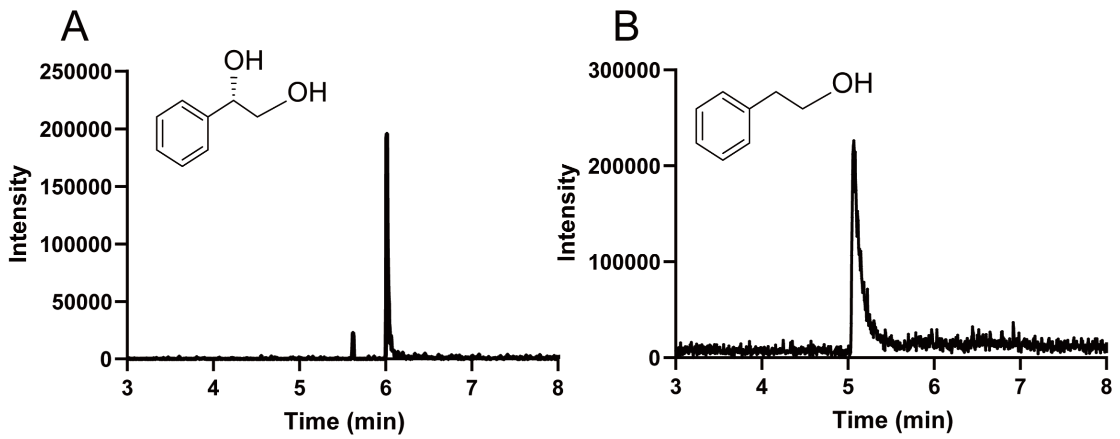

Figure S7. GC-MS detection of (A) (*S*)-PED after mixing modules 1 and 2 and (B) 2-PE after mixing modules 1 and 3.
